# Exploring the Interplay of Dataset Size and Imbalance on CNN Performance in Healthcare: Using X-rays to Identify COVID-19 Patients

**DOI:** 10.3390/diagnostics14161727

**Published:** 2024-08-08

**Authors:** Moshe Davidian, Adi Lahav, Ben-Zion Joshua, Ori Wand, Yotam Lurie, Shlomo Mark

**Affiliations:** 1 Guilford Glazer Faculty of Business and Management, Ben-Gurion University of the Negev, Beer-Sheva 8410501, Israel; yotam@som.bgu.ac.il; 2 Software Engineering Department, SCE—Shamoon College of Engineering, Beer-Sheva 84100, Israel; adilahav82@gmail.com; 3 Department of Otorhinolaryngology, Barzilai University Medical Center, Ashkelon 7830604, Israel; benzionj@bmc.gov.il; 4 Division of Pulmonary Medicine, Barzilai University Medical Center, Ashkelon 7830604, Israel; oriw@bmc.gov.il; 5 Faculty of Health Sciences, Ben-Gurion University of the Negev, Beer-Sheva 8410501, Israel; 6 Software Engineering Department, SCE—Shamoon College of Engineering, Ashdod 77245, Israel; marks@sce.ac.il

**Keywords:** CNN, imbalanced data, classification biases, COVID-19, pulmonary disease

## Abstract

Introduction: Convolutional Neural Network (CNN) systems in healthcare are influenced by unbalanced datasets and varying sizes. This article delves into the impact of dataset size, class imbalance, and their interplay on CNN systems, focusing on the size of the training set versus imbalance—a unique perspective compared to the prevailing literature. Furthermore, it addresses scenarios with more than two classification groups, often overlooked but prevalent in practical settings. Methods: Initially, a CNN was developed to classify lung diseases using X-ray images, distinguishing between healthy individuals and COVID-19 patients. Later, the model was expanded to include pneumonia patients. To evaluate performance, numerous experiments were conducted with varied data sizes and imbalance ratios for both binary and ternary classifications, measuring various indices to validate the model’s efficacy. Results: The study revealed that increasing dataset size positively impacts CNN performance, but this improvement saturates beyond a certain size. A novel finding is that the data balance ratio influences performance more significantly than dataset size. The behavior of three-class classification mirrored that of binary classification, underscoring the importance of balanced datasets for accurate classification. Conclusions: This study emphasizes the fact that achieving balanced representation in datasets is crucial for optimal CNN performance in healthcare, challenging the conventional focus on dataset size. Balanced datasets improve classification accuracy, both in two-class and three-class scenarios, highlighting the need for data-balancing techniques to improve model reliability and effectiveness. Motivation: Our study is motivated by a scenario with 100 patient samples, offering two options: a balanced dataset with 200 samples and an unbalanced dataset with 500 samples (400 healthy individuals). We aim to provide insights into the optimal choice based on the interplay between dataset size and imbalance, enriching the discourse for stakeholders interested in achieving optimal model performance. Limitations: Recognizing a single model’s generalizability limitations, we assert that further studies on diverse datasets are needed.

## 1. Introduction

### 1.1. Classification Biases in CNN Due to Imbalanced Data

One of the prominent applications of Machine Learning (ML) systems revolves around the task of classifying items into appropriate categories. Classification entails the utilization of algorithms that acquire the ability to assign labels to instances within a given problem domain. However, in many instances where a system is tasked with learning how to classify, there exists substantial dissimilarity in the representation of each class within the training data, commonly referred to as class imbalance. This issue has become a significant challenge within the ML community in recent years. Class imbalance occurs when the number of instances belonging to one class (referred to as the majority class) far exceeds the number of instances belonging to the other class (known as the minority class) [1]. Consequently, this imbalance in class distribution poses difficulties in accurately classifying the minority instances, thereby resulting in a degradation of overall performance. Furthermore, it is essential to note that the identification of the minority class is often of greater interest, as the misclassification of these instances incurs higher costs [2].

The issue of data imbalance is a pervasive challenge encountered across various domains, including, but not limited to, credit card fraud detection, spam email classification, computer and network security against hacking, and other scenarios involving anomaly detection. The prevalence of this problem across diverse fields underscores its critical importance in data analytics and ML. In healthcare, the problem of imbalanced data is particularly significant, especially when employing ML classification systems to categorize patients into two groups: “Sick” and “Healthy”. The primary objective of such systems is to accurately identify the “Sick” class, which, in most cases, comprises the minority class, in relation to the larger “Healthy” class [3]. Class imbalance in healthcare data can lead to the biased performance of ML models, favoring the majority class while neglecting the minority classes [4,5] (the antithesis of the intended objective).

Researchers and practitioners have developed various strategies and methodologies to address the challenge of imbalanced data. These include data augmentation, such as a study that proposed a method involving the generation of synthetic samples to increase the size of the training dataset, thereby mitigating imbalanced data related to asthma [6]. Another study applied image augmentation to address imbalanced data problems in cervical cancer diagnosis using Pap smear images [7]. Data-balancing techniques, such as the combination of under-sampling and instance selection methods, have been demonstrated in diagnosing heart failure [8]. Additionally, the Random Over-Sampling algorithm has been employed in research on imbalanced electrocardiogram (ECG) data analysis [9], as well as in the analysis of surface electromyography (sEMG) data for distinguishing between normal and abnormal subjects in knee studies [10]. Advanced models, such as Generative Adversarial Networks (GANs) and long short-term memory (LSTM) networks, have been explored to overcome imbalanced data challenges and improve the diagnosis of heart diseases [11]. Furthermore, specialized methodologies like imbalance-aware nuclei segmentation have been developed to address class imbalance issues in analyzing Hematoxylin and Eosin stained histopathology images [12]. and overcome imbalanced data challenges at the pixel level using a hybrid loss function aiming to detect differential patterns for diagnosing pigmented skin lesions [13].

The existing literature generally agrees that data imbalance has a detrimental effect on the performance of classification systems [14,15]. However, there is no consensus regarding the threshold at which data imbalance begins to impact performance or the extent of its influence. Technically, any dataset that exhibits an unequal distribution between groups can be categorized as imbalanced. Nevertheless, the prevailing understanding in the research community is that imbalanced data refers to those datasets that demonstrate a significant imbalance, and, in certain cases, an extreme one. Conversely, there are studies that argue that even a minor imbalance in training data can lead to a deterioration in classification performance [14,16]. In addition, most of the research carried out into imbalanced classes mainly focuses on binary classification [17]. This is despite the fact that, in most applications where classification is needed, there are more than two groups [18].

Our article focuses explicitly on CNN, a specific model of ML designed for processing structured data like images. We explore how dataset size and imbalance levels influence CNN performance. Generally, larger datasets tend to impact ML models’ performance positively [19]. With an increased volume of data, the models have more opportunities to learn and generalize patterns, leading to improved accuracy and robustness. Despite this, some studies claim that improving system performance is not necessarily a result of increasing the dataset [20,21]. In addition, larger datasets may come with increased computational and storage requirements, which should be carefully considered in practical applications. Hence, a judicious choice of dataset size is vital for achieving optimal performance in ML systems.

Consider the following scenario: a dataset comprising information from 100 patients prompts a question on the superiority between two alternatives: utilizing a dataset with a total size of 200 (consisting of 100 patients and 100 healthy individuals) versus employing a dataset with a total size of 500 (comprising 100 patients and 400 healthy individuals). On the one hand, the volume of data in the former option is smaller than in the latter. Conversely, the former option presents a balanced dataset, in contrast to the latter option, with a balance ratio of 20:80 between patients and healthy individuals, respectively. The inquiry regarding the superiority between the two dataset options will be addressed subsequently as part of the study.

This study aims to make significant contributions to the understanding and optimization of ML systems for classification tasks in the context of healthcare applications. By thoroughly investigating the effects of dataset size and data imbalance, it seeks to shed light on the nuanced relationship between these factors and the resultant impact on performance, both for the binary case and for the case where there are three classification groups. The study’s findings will help define the critical thresholds for data imbalance beyond which performance degradation occurs, providing valuable insights for data preprocessing and balancing techniques. Moreover, the study’s examination of the effect of dataset size on system performance will contribute to the development of guidelines for selecting appropriate dataset sizes, considering computational and storage constraints while achieving optimal model performance. Ultimately, the study endeavors to provide practitioners and researchers in the field of healthcare ML with evidence-based guidance on how to best utilize and optimize datasets, thereby fostering more robust and effective ML systems for healthcare applications.

### 1.2. Overcoming Data Challenges in CNNs

Addressing data imbalance is crucial for enhancing the performance of CNNs in healthcare applications. Although this research does not deal with data-balancing methods and focuses on the effect of the training set on system performance, we decided to provide a brief overview of central approaches in the literature to deal with this challenge.

The first approach is advanced neural network architectures such as Deep Residual Networks (ResNets) [22]. ResNets introduce skip connections or shortcuts to jump over some layers. This architecture helps train deep networks by mitigating the vanishing gradient problem. ResNets have shown promise in handling large and imbalanced datasets by allowing the network to learn residual functions instead of unreferenced functions, improving overall model performance and generalization. Another example is Dense Convolutional Networks (DenseNets) [23]. DenseNets connect each layer to every other layer in a feed-forward fashion. These networks enhance information flow and gradient propagation, making them effective for working with limited and imbalanced data. The dense connections reduce the risk of overfitting, especially when dataset sizes are constrained.

The second approach involves hybrid models such as CNN-RNN Hybrids. Combining CNNs with Recurrent Neural Networks (RNNs), such as long short-term memory (LSTM) networks, can be advantageous [24]. While CNNs are excellent at spatial feature extraction, RNNs, particularly LSTMs, effectively capture temporal dependencies. This combination can be helpful in medical imaging applications where sequential or time-series data are involved, improving the robustness of the model against data imbalance [25]. Another example is Generative Adversarial Networks (GANs). GANs can generate synthetic data to augment the dataset, addressing the imbalance issue. A GAN comprises a generator and a discriminator that are trained simultaneously. The generator creates synthetic data that are as realistic as possible, while the discriminator attempts to distinguish between natural and synthetic data. This method can help create a balanced dataset without losing the diversity and complexity of the original data.

The third approach includes ensemble methods such as ensemble learning. Ensemble learning that combines multiple models, such as Bagging (Bootstrap Aggregating) and Boosting [26,27], can help improve the performance of classifiers on imbalanced datasets. Techniques like AdaBoost and Gradient Boosting build strong classifiers by focusing on difficult-to-classify instances, thus enhancing the model’s ability to learn from minority-class samples. Another example is stacking [27]. Stacking involves training multiple models and using their predictions as inputs to a meta-classifier, which makes the final prediction. This approach leverages the strengths of different models and can be particularly effective in handling data imbalance by ensuring that diverse perspectives on the data are considered.

The fourth approach is algorithm-level, which concentrates on improving the ability of the classifier [28], for example, cost-sensitive learning methods such as weighted loss functions. Implementing weighted loss functions that assign higher penalties to misclassifications of minority class samples can help the model pay more attention to underrepresented classes. Another example is class-balanced loss, which considers the effective number of samples in each class and adjusts the loss contribution from each class based on its representation in the dataset. This method ensures that minority classes have a more significant impact on the model’s learning process.

The last approach is sampling methods, which address data imbalance by manipulating the distribution of data in the training datasets. Techniques such as under-sampling (reducing the number of majority class samples), over-sampling (increasing the number of minority class samples), and hybrid methods (combining both approaches) aim to balance class distributions and improve model performance [28].

By leveraging these approaches, researchers can address the limitations posed by data imbalance and dataset size, thereby improving the performance and generalizability of CNNs in healthcare diagnostics.

### 1.3. Medical Background

The term “pulmonary disease” encompasses a spectrum of disorders that hinder the normal functioning of the respiratory system. Among these, infectious diseases represent prevalent and clinically significant respiratory pathologies, often attributed to a diverse array of bacterial, viral, or fungal pathogens. Additionally, the respiratory system can be affected by other ailments such as asthma, occupational lung diseases resulting from occupational exposures, and the formidable challenge posed by lung cancer.

COVID-19, officially known as Coronavirus Disease 2019, is an infectious illness caused by the novel coronavirus Severe Acute Respiratory Syndrome Coronavirus 2 (SARS-CoV-2), which was initially identified in China towards the end of 2019. Since its emergence, the virus has rapidly spread across the globe, prompting the World Health Organization to declare it a pandemic on 11 March 2020 [29]. The symptoms of COVID-19 are diverse, but commonly include fever, cough, headache, fatigue, gastrointestinal distress, and the loss of smell and taste [30]. The disease primarily affects the respiratory system, often leading to pneumonia and respiratory failure [31]. Reverse-Transcriptase Polymerase Chain Reaction (RT-PCR) tests, which detect viral RNA, are considered the recommended diagnostic method for COVID-19 due to their high sensitivity and specificity in detecting infections. However, RT-PCR tests are relatively costly, require specialized equipment and trained personnel, cannot be performed at the point of care, and may take more than 24 h to deliver results. In contrast, rapid antigen detection tests, particularly lateral-flow assays, offer quicker results within minutes, are more affordable, and do not necessitate laboratory facilities or extensive human resources. Nevertheless, their lower detection limit results in reduced sensitivity compared to molecular RT-PCR. Both types of tests have been associated with false negative results, which can occur due to factors such as low viral load, errors in specimen collection, technical difficulties during testing, and other variables, despite the presence of positive clinical and radiological findings [32].

Chest imaging using computed tomography (CT) has demonstrated the ability to reveal characteristic radiographic features in nearly all individuals affected by COVID-19. Some studies have even suggested that CT scans may exhibit greater sensitivity compared to RT-PCR in the diagnosis of COVID-19 [33]. Specifically, in patients with suspected COVID-19 infection, the reported sensitivity of chest CT for detecting COVID-19 infection was 86.9%, accompanied by a specificity of 78% [34].

**Figure 1:**
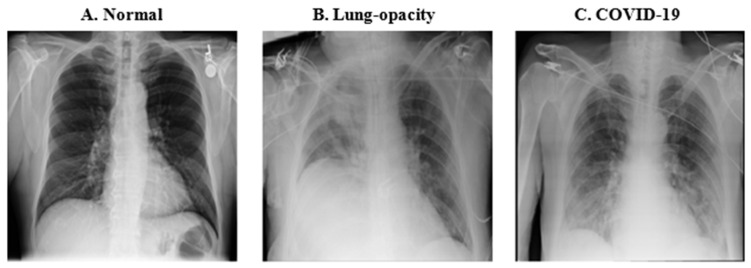
Chest X-ray figures used for the training. (**A**). (Normal fig.) A normal chest X-ray of a healthy individual. (**B**). (Lung-opacity fig.) A patient with unilateral right-sided opacity from another cause. (**C**). (COVID-19 fig.) A COVID-19 patient with bilateral opacities consistent with COVID-19 pneumonia.

The most prevalent radiological manifestations of lung infection in individuals with COVID-19 consist of consolidations and ground-glass opacities. These radiographic features typically exhibit a bilateral distribution, with a predilection for the lower lobes and peripheral regions of the lungs. It is important to note that these findings, along with other patterns such as diffuse lung opacities, are not exclusively indicative of COVID-19 and can be present in other pulmonary conditions [35]. Chest X-rays offer a convenient, cost-effective, and rapid imaging modality for assessing individuals suspected of COVID-19 infection, with portable X-ray machines being widely employed in such scenarios. Figure 1 provides a visual comparison of chest X-rays depicting a healthy individual and a COVID-19 patient with evident lung opacity.

## 2. Methods

The initial phase of our study involved constructing a CNN model, a specific ML system application capable of detecting COVID-19 patients using chest X-ray images. This model served as a tool to investigate the influence of imbalanced training data on system performance. Its purpose was to classify individuals into two categories: healthy or COVID-19 patients, and subsequently include patients with pneumonia [36]. Within this context, the COVID-19 class represents the minority class compared to healthy individuals (referred to as “Normal”). Upon achieving a highly accurate model, we proceeded to the second phase of our study, which aimed to examine the impact of different levels of data imbalance on system performance. Additionally, we sought to assess how the size of the dataset affected system performance and the imbalance.

The imbalance ratio (IR) refers to the quantitative measure of class imbalance in a dataset and is calculated as the ratio of the number of instances in the majority class to the number of instances in the minority class [2]. In the context of our current study, our investigation focuses on analyzing the impact of various IR levels on the performance of a classifier that we have developed. By systematically examining different IR levels, we aim to gain insights into how class imbalance affects the classifier’s effectiveness and overall performance.

### 2.1. The Classifier

The classifier utilized in this study is a CNN model—a deep learning algorithm that possesses the ability to analyze input images, discern the significance of different image features, and accurately differentiate between distinct elements within the image. Specifically, in our study, the CNN model is employed to examine chest X-ray images, with the objective of performing a classification task to accurately identify whether a patient exhibits a healthy lung condition or is afflicted with a lung disease. Through the utilization of the CNN model, we aim to achieve accurate and reliable classification outcomes for the given diagnostic task.

### 2.2. Convolutional Neural Network (CNN) Architecture

Input and Preprocessing—The input to the CNN is X-ray images resized to 224 × 224 pixels. This resizing ensures uniformity across the dataset and compatibility with the network architecture.

Network Architecture—The CNN architecture consists of five convolutional layers, each followed by specific operations to enhance feature extraction and reduce dimensionality:

1. Convolutional Layers: There are five convolutional layers, each responsible for extracting features from the input images.
  - First to Fourth Convolutional Layers: Each of these layers is followed by a Max Pooling layer to downsample the feature maps, reducing the spatial dimensions while retaining important features.
  - Fifth Convolutional Layer: This layer is followed by a Batch Normalization layer to stabilize and accelerate the training process.
2. Flattening Layer: After the convolutional layers, the output is flattened into a one-dimensional array to be fed into the fully connected layers.
3. Dense Layer: The flattened output is passed through a dense layer, followed by another Batch Normalization layer to further stabilize training.
4. Dropout Layer: A Dropout layer is employed to prevent overfitting by randomly setting a fraction of the input units to zero during training.
5. Output Layer: The network ends with a dense output layer.
  - For binary classification (COVID-19 positive vs. negative), the output layer contains two neurons.
  - For multi-class classification (e.g., COVID-19, Pneumonia, Normal), the output layer contains three neurons.

**Figure 2:**
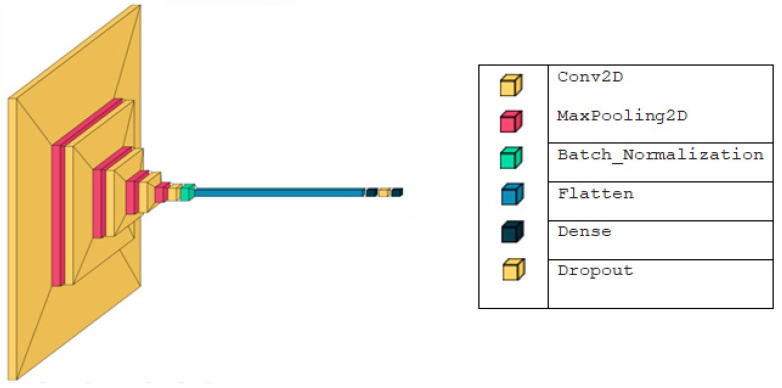
CNN architecture—Figure illustrating the detailed architecture of the CNN, as described above.

Parameter Details—The specific parameters for each layer, including filter size, stride, and activation functions, are detailed in Table 1 and illustrated in Figure 2.

Weight Initialization and Consistency—To ensure consistency across experiments, initial weights for the network were randomly generated and saved. These saved weights were then used to initialize the network before each experiment, ensuring all runs started from the same initial state.

Training and Optimization—The CNN model was trained for a fixed number of epochs—20 epochs per run. This number was determined after experimenting with up to 60 epochs, where no significant improvement in performance was observed beyond 20 epochs. The epoch with the highest classification accuracy was selected as the best model.

Performance Metrics—The model’s performance was evaluated based on classification accuracy, sensitivity, specificity, precision, recall, F1-score, and G-measure. These indices provide a comprehensive understanding of the model’s effectiveness in classifying the X-ray images correctly. An explanation of the indices can be found in Supplementary Material File S1.

### 2.3. Comparison with Other Networks for COVID-19 Classification

In the study by Nur et al. [37], a comprehensive review of approximately 20 articles is provided, focusing on the development of networks designed to classify COVID-19 using various datasets. The performance of these networks, as measured by accuracy, ranged between 71.9% and 100%. Notably, only one network achieved an accuracy of 100%, and this was accomplished by combining three distinct networks: AlexNet, GoogleNet, and ResNet18.

Our developed CNN network achieved an accuracy of 97.6%, which is competitive within the range reported by Nur et al. Despite this, we did not pursue further enhancements to push our network’s performance closer to the 100% mark. This decision was guided by two primary considerations. First, we intentionally selected a specific network architecture to achieve an accuracy lower than 100%. Achieving an accuracy very close to 100% would have limited our ability to explore the impact of different balance ratios on model performance. If our network had reached near-perfect accuracy, it would have been challenging to discern whether varying the balance ratio could yield any further improvements. By maintaining a slightly lower accuracy, we preserved the sensitivity of our model to changes in data balance, allowing for a thorough investigation into how different levels of imbalance affect performance. Second, to conduct the extensive series of experiments required for our study, it was essential to utilize a network architecture with a relatively low number of layers. Our network only comprises 15 layers, which contrasts significantly with the ResNet18 network, which has approximately 70 layers. The simpler architecture of our network ensured that we could efficiently run hundreds of experiments without incurring prohibitive computational costs or extended processing times.

### 2.4. Data Description

The dataset utilized in this study comprises a collection of chest X-ray images, which are categorized into three distinct classes: healthy individuals, COVID-19 patients, and individuals with lung opacity. The distribution of the data within each class is as follows:

1. Normal: The dataset consists of 10,192 images, which were further augmented by horizontally inverting the images, resulting in a final count of 10,500 images.
2. COVID: The dataset encompasses 3616 images specifically related to COVID-19 cases.
3. Lung Opacity: The dataset contains 6012 images of individuals displaying lung opacity.

The chest X-ray images utilized in this study were sourced from the COVID-19 Radiography Database [38], a recognized repository for medical imaging data related to COVID-19 cases.

To maintain the generalizability of our study, we minimized the use of data augmentation techniques. Our objective was to explore the impact of dataset size and imbalance on CNN performance without the influence of extensive data augmentation methods. Consequently, data augmentation was deliberately avoided except in specific instances.

The only scenario where data augmentation was applied involved the Normal class during tests with a 10K dataset. Given that the Normal class originally consisted of 10,192 images, a minor augmentation was necessary to reach a dataset size of 10,500 images for sufficient training and testing. In this case, we utilized the horizontal inversion of images to achieve the required dataset size.

This controlled use of data augmentation ensures that our findings remain broadly applicable and not restricted by augmentation-specific factors.

### 2.5. Split Data

Two distinct options were considered for splitting the dataset into training, validation, and testing subsets:

1. “Test 20%” This widely employed approach for training neural networks involves dividing the data in a 70:10:20 ratio. Consequently, 70% of the data is allocated for training, 10% for validation purposes, and 20% for testing. Alternatively, this option can be viewed as an 80:20 split, as 80% of the data is used for assessing and optimizing the model’s performance through training and validation, while the remaining 20% is utilized to evaluate the model’s performance.
2. “Test 500”: In addition to the aforementioned option, an alternative approach was adopted, wherein the data were divided into two distinct portions. The first portion was employed for training and validation in an 80:20 ratio, respectively. The second portion was reserved exclusively for testing the model’s performance and included 500 chest X-ray images for each class. The selection of 500 images per class was aimed at achieving a level of accuracy to three decimal points.

Furthermore, two different methods were employed to manipulate the imbalance ratio (IR) between classes:

1. “Fix”: The number of images in the minority class (COVID-19) remained fixed, while the number of images in the majority class was systematically varied during each experiment. The experimentation began with a balanced ratio (50:50) and concluded with a significantly high IR (1:99).
2. “Change”: In this approach, the number of images in the minority class was deliberately reduced, while an equivalent increase in the number of images within the majority class was made.

These modifications in the dataset’s class distribution allowed for an exploration of the impact of varying IR levels on the performance of the classification model.

### 2.6. Imbalance Ratio

The performance of the model was evaluated by varying the imbalance ratios (IRs) between classes. Our investigation primarily focused on IRs ranging from 50:50 to 1:99, as any ratios exceeding 1:100 were considered imbalanced data that could potentially reduce performance. The distribution of data among the classes is provided in Table 2. Additionally, we examined the impact of dataset size on the model’s performance, considering three different dataset sizes: 1000, 5000, and 10,000 images. Furthermore, the model was assessed under different scenarios where the minority class comprised 100, 200, and 500 images.

### 2.7. Experiments

In order to assess the performance of the model, a range of indicators were utilized for the quantitative analysis of the classification performance, as detailed in Supplementary Material File S1.

For the binary classification task (COVID vs. Normal), approximately a hundred simulation experiments were conducted to investigate the impact of class imbalance on the classification model’s performance. Table 3 provides an overview of all tests performed for binary classification, and the results of these experiments can be found in Supplementary Material File S2 (Tables S1–S12).

Regarding the classification of three classes (COVID vs. Normal vs. Lung Opacity), a data splitting method was employed where the amount of training data for each class varied (Change), while maintaining a constant sum of data across all classes. Table 4 summarizes the experiments conducted for the three-class classification, and the results of these experiments can be found in Supplementary Material File S2 (Tables S13–S18 and Figures S1–S6).

## 3. Results

**Figure 3:**
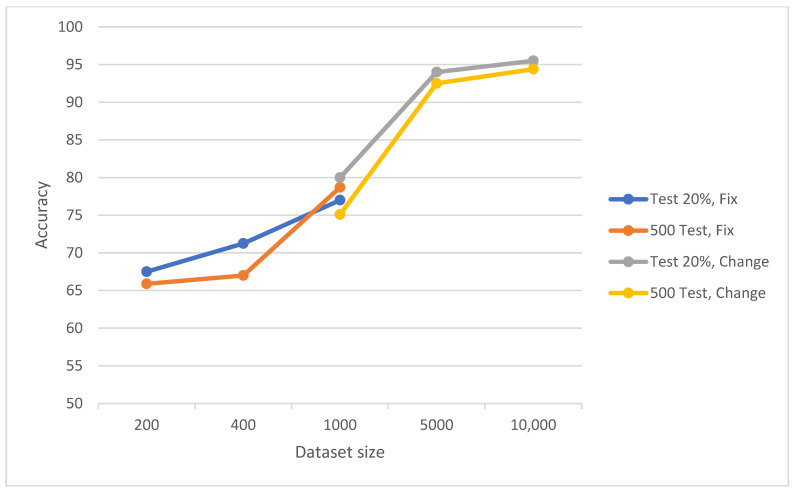
Consistent patterns across all four data-split methods utilized. As the training set size progressively increases, there is a corresponding improvement in the level of accuracy.

Our study employed various evaluation techniques for the classification model, including manipulating the dataset size, adjusting the imbalance ratio of class samples, and modifying the distribution of the dataset among training, validation, and testing. In the case of binary classification, when working with a balanced dataset, the results revealed consistent patterns across all four data-split methods utilized. As the size of the training dataset increased, enhancements in the system’s accuracy were observed, as depicted in Figure 3. For instance, when combining the “Test 20%” method with the “Change” approach, the accuracy rose from 80% for a training data size of 1000 images to 94% for 5000 images. Similarly, when employing the “Test 500” method in conjunction with the “Change” technique, the accuracy increased from 75.1% for a training data size of 1000 images to 92.5% for 5000 images.

**Figure 4:**
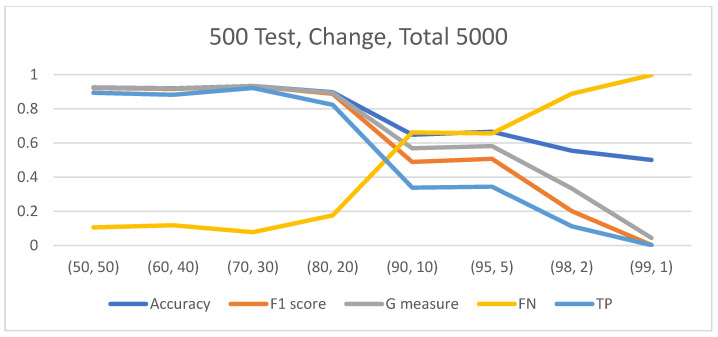
An increase in the IR led to decreased accuracy.

**Figure 5:**
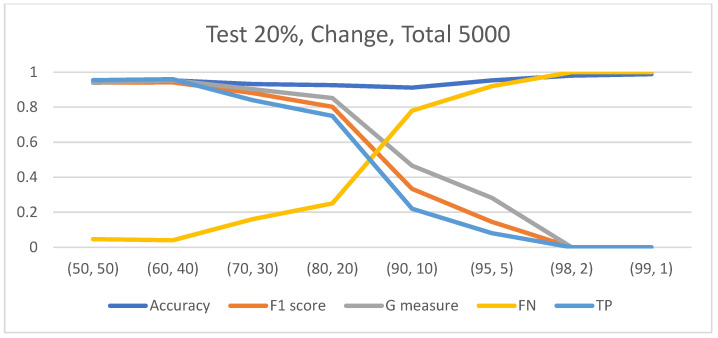
At an IR of 1:99, although the model failed to classify any instances from the minority class correctly, the overall accuracy reached 98.9%.

In the case of an imbalanced dataset, regardless of the data-split method employed, the results consistently demonstrated that as the imbalance ratio (IR) between classes increased, the classification model tended to label more images as belonging to the majority class. It is noteworthy that when the IR reached an extreme level, such as 1:99, the model exhibited significant difficulties in correctly classifying instances from the minority class. However, when examining the accuracy metric, two distinct patterns emerged. With the “Test 500” data-split method, an increase in the IR led to decreased accuracy (Figure 4). Conversely, in the context of the “Test 20%” data-split method, a discernible trend emerged: as the IR varied from 4:6 to 1:9, there was a minor decline in accuracy. However, beyond an IR of 1:9, the accuracy exhibited a more substantial ascent, culminating in an accuracy level of 98.9% at an IR of 1:99. At an IR of 1:99, although the model failed to classify any instances from the minority class correctly (sensitivity = 0%), the overall accuracy reached 98.9% (Figure 5). The behavior observed with the “Test 20%” method can be explained by the fact that at an IR of 1:99, the majority class constituted 99% of the data. Since the test data size is directly proportional to the dataset size (20%), 99% of the test data consisted of instances from the majority class. Consequently, the model achieved perfect classification of the majority class (specificity = 100%) but failed to correctly classify the minority class, which comprised only 1% of the data (sensitivity = 0%), resulting in a high accuracy of 99%. It is important to note that across all classification methods, unlike accuracy, the F1 score and G measure demonstrated a decline in the model’s ability to accurately classify instances as the IR increased, as evidenced by the accuracy measure using the “Test 500” method.

**Figure 6:**
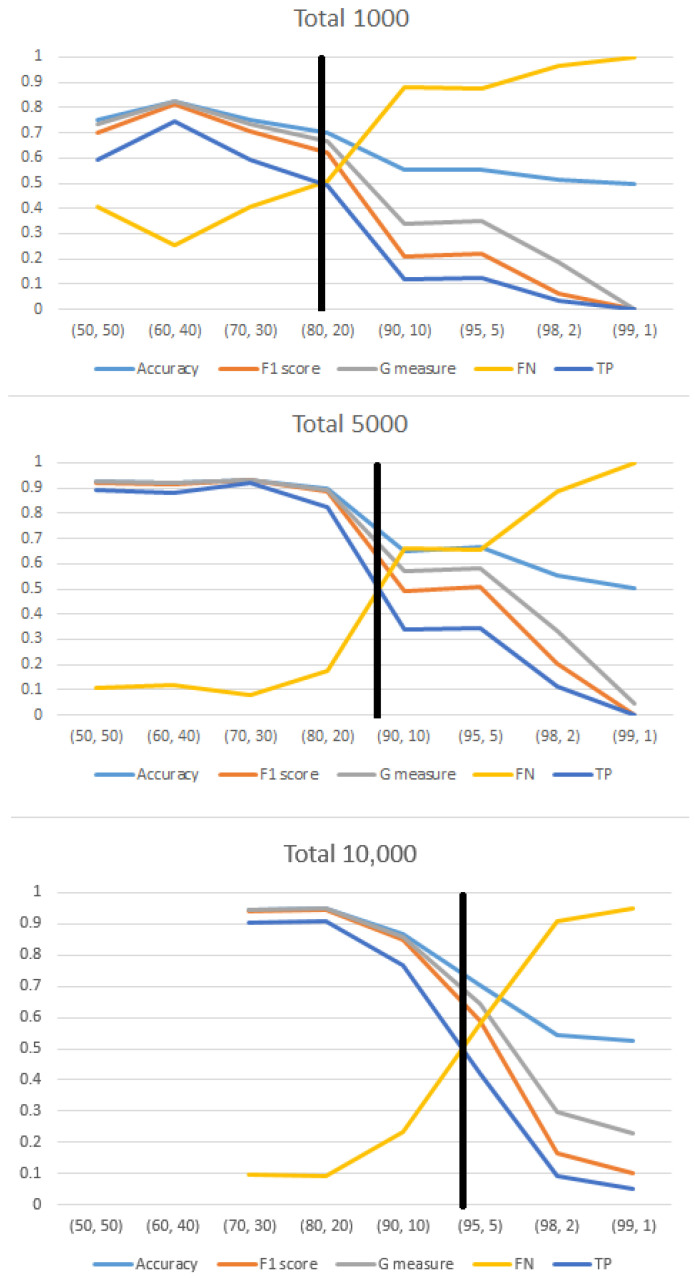
As the size of the training set increases, the point at which the model’s sensitivity drops below 50% occurs at more extreme imbalance ratios. The black line on the graph represents the sensitivity threshold dropping below 50%. For a 1000-image training set, this occurs around an IR of 20:80. With a 5000-image training set, the threshold is slightly before an IR of 10:90. For a 10,000-image training set, the threshold is just before an IR of 5:95.

In order to investigate the impact of imbalance on the classification performance, particularly on the sensitivity of the minority class, we employed the “Test 500” method combined with the “Change” approach. Our findings revealed that the dataset size played a moderating role in influencing the sensitivity of the system in the presence of imbalance (Figure 6). Specifically, with a training set consisting of 1000 images, a decline in sensitivity became apparent at an imbalance ratio (IR) of 40:60. For a larger training set of 5000 images, a significant decrease in sensitivity was observed between an IR of 20:80 (82.4%) and an IR of 10:90 (33.8%). Moreover, when the training set contained 10,000 images, a decrease in sensitivity was observed between an IR of 20:80 (90.8%) and an IR of 10:90 (76.6%), and a more pronounced decrease was observed with an IR of 5:95 (42.2%). These results clearly indicate that a larger amount of data (images) mitigates the impact of imbalance on the system’s performance.

We shall delve into the inquiry posited in the Introduction concerning the comparative advantages between two distinct datasets: a size-200 set consisting of 100 patients and 100 healthy individuals, and a size-500 set comprising 100 patients and 400 healthy individuals. Two methods, Test 20% and Test 500, were used to evaluate these options. The results indicate that when the dataset is balanced, both metrics perform better than the larger unbalanced dataset: Test 20% with an F1 score of 0.648 and a G index of 0.67, and Test 500 with an F1 score of 0.645 and a G index of 0.657. Conversely, the F1 scores and G index for the larger but unbalanced dataset were significantly lower, with values of 0.083 and 0.219 for Test 20%, and 0.423 and 0.517 for Test 500. However, we did not obtain a uniform result for accuracy. Test 20% indicated that the second option was superior (despite its unbalanced nature) with an accuracy of 78% compared to 67.5% for the balanced dataset. While Test 500 demonstrated the reverse, the first option was superior, with a higher accuracy of 65.9% for the smaller but balanced dataset compared to 61.1% for the larger but unbalanced dataset. Thus, in the examined case, the balanced dataset with a size of 200 demonstrated enhanced performance when compared to the unbalanced dataset with a size of 500.

**Figure 7:**
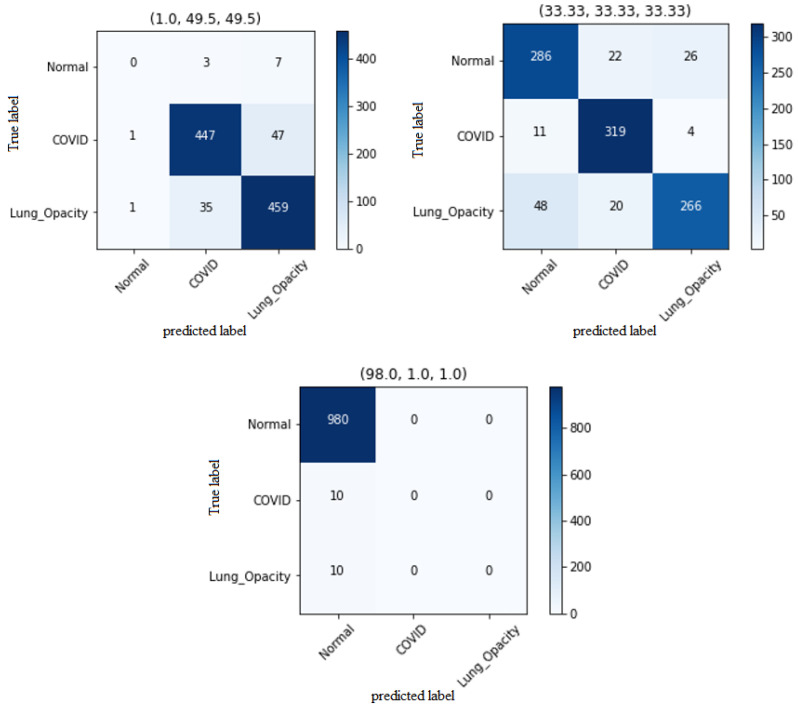
As the percentage of the data of a class decreased, the model’s ability to classify that class also decreased. In the balanced dataset, the count of accurately classified images was comparably consistent across each class. However, as the class imbalance intensified, the model tended to classify more images into the majority class. With an extreme imbalance ratio of 1:1:98, the model demonstrated precise classification of the majority class while misclassifying the minority classes.

The behavior of three-class classification exhibited similarities to binary classification. In both the Test 20% and Test 500 data-split methods, a clear correlation was observed between the representation percentage of a specific class in the dataset and the model’s classification performance for that class. As the percentage of a class decreased, the model’s ability to classify that class, indicated by sensitivity, also decreased (Figure 7). In the case of a balanced dataset with equal representation of all three classes, similar sensitivity values were observed for each class. However, as the imbalance between the classes increased, the model tended to classify more images as belonging to the majority class. Notably, with an extreme imbalance ratio of 1:1:98, the model demonstrated accurate classification of the majority class and misclassification of the minority classes for every image. Furthermore, in the three-class classification scenario, the results demonstrated that the “Macro avg F1 score” served as an indicator that effectively reflected the model’s ability to classify the minority classes, without being overly influenced by the classification of the majority class.

## 4. Discussion

This study reveals a positive correlation between dataset size and performance metrics, F1, and G-measure, indicating that larger datasets lead to improved performance in ML models, as claimed by Xiaolong et al. [19]. However, a noteworthy observation is that the improvement in performance exhibits a saturation phenomenon as the dataset size exceeds a certain threshold, specifically when the dataset contains more than 5000 samples. Beyond this point, there is no consistent and proportional increase in performance, suggesting that the models’ learning capacity reaches a plateau, which can explain the claims that the dataset size does not necessarily affect the model’s performance [20,21]. Consequently, additional data beyond the saturation point do not yield a consistent overflow in performance gains, implying that the benefits of further increasing the dataset size become limited and may not justify the associated resource costs.

The study also demonstrates that balanced datasets with equal representation of different classes allow the models to learn and generalize more effectively, avoiding biases towards the majority class. This finding corroborates the existing literature that highlights the negative influence of data imbalance on classification systems and further emphasizes the significance of balancing the data to achieve better performance in healthcare applications. Based on the test case in this paper, the threshold at which data imbalance starts to affect performance depends on the size of the dataset: the larger the dataset, the smaller the impact of the imbalance.

An intriguing and novel result of this study is the observation that the effect of balance ratio in the data has a more significant impact on performance than the effect of dataset size. In other words, while increasing the dataset size positively influences performance, achieving a balanced representation of classes in the dataset has a greater influence on the model’s ability to classify accurately. This finding challenges conventional wisdom, which often prioritizes dataset size in ML applications, and highlights the crucial role of data-balancing techniques in enhancing classification performance.

The aforementioned trend is also evident in the specific case described at the end of the introduction. The study’s outcomes reveal that, when the dataset is balanced, the ML system performs more favorably compared to the larger dataset that is unbalanced. This observation corroborates the broader finding regarding the impact of dataset size on performance and aligns with the notion that achieving class balance within the dataset yields better results, even if the dataset size is relatively smaller.

These findings have important practical implications for the development of ML systems in healthcare. In scenarios with limited data availability, it is advisable to focus on balancing the dataset, even if that results in a smaller dataset size, as it can lead to more substantial improvements in performance. Additionally, researchers and practitioners should consider using appropriate data-balancing methods to mitigate the negative effects of data imbalance and improve model accuracy and reliability. Moreover, while increasing the dataset size remains beneficial, the study emphasizes that achieving balance should be a primary consideration to optimize system performance in healthcare applications.

### CNN Performance Saturation beyond Dataset Size

The performance of CNNs is a critical factor in their successful application in various fields, including healthcare diagnostics. As we saw in the research results, increasing the size of the training set improves the performance of the CNN model. Still, there is a saturation point beyond which additional data yield diminishing returns. Understanding the underlying reasons for this saturation is essential to optimizing model training and resource allocation. In this chapter, we will review several reasons for this phenomenon.

First, after a certain point, the additional data contribute less to the learning process because the model has already captured most of the underlying patterns and features present in the data. Each new data point provides less new information to the model in this phenomenon. One of the reasons for this is that large datasets often have a high degree of redundancy, meaning that many data points are similar or repetitive. When the model is exposed to redundant data, the incremental benefit of each additional data point decreases. Another reason is that CNNs have a finite capacity to learn and generalize from data. The network architecture, including the number of layers and parameters, determines this capacity. Once the model has effectively utilized its capacity to learn the patterns in the data, additional data may not significantly improve performance because the model has already reached its learning potential.

Second, once a critical mass of data is reached, the model has likely learned to generalize well, and additional data reinforce what it has already been learned without providing new challenges or information. A less common cause is that if the additional data include a higher proportion of noisy or irrelevant information, they can dilute the quality of the learning signal, leading to a plateau or even a decrease in performance.

Beyond all of the above, as datasets grow, the computational resources required for training also increase. At some point, the cost of training with additional data may outweigh the performance benefits. The marginal gains in performance may not justify the time and resources needed to process more data.

In summary, while increasing the dataset size generally improves the performance of CNNs, there is a saturation point where the benefits of additional data diminish. This occurs due to diminishing returns in learning, the finite capacity of the model, data redundancy, overfitting prevention, computational limits, and the introduction of noise. Understanding these factors helps optimize the use of data and resources for training CNN models.

## 5. Conclusions

This study investigated the influence of dataset size, data imbalance, and their interplay on the performance of CNN systems for image classification, particularly for diagnosing diseases using lung X-ray images. The results demonstrated that larger datasets generally improve system accuracy, although this improvement reaches a saturation point beyond which performance gains diminish.

A unique aspect of this study is its examination of a three-class classification scenario, a case often overlooked in other studies. The findings revealed a clear correlation between the representation percentage of a class in the dataset and the model’s classification performance for that class. As the representation of a class decreased, the model’s sensitivity for that class also reduced. Balanced datasets, with equal representation of all classes, yielded similar sensitivity values for each class. However, as class imbalance increased, the model tended to classify more images as belonging to the majority class, highlighting the critical role of dataset balance in enhancing system performance.

The study underscored the importance of addressing data imbalance to achieve better classification results. Balanced datasets outperformed larger unbalanced datasets, with the balance ratio having a more pronounced impact on performance than dataset size. Imbalance introduced bias into the system’s performance, causing a tendency to classify more examples into the majority group, with the severity of this bias increasing with greater imbalance.

The study also highlighted the importance of maintaining a balanced distribution of test data between different classes and selecting appropriate performance measures. In imbalanced datasets, a balanced distribution of test data is essential for obtaining reliable performance measurements, and measures like F1 score and G-Mean are preferable for accuracy.

The findings from this study contribute to a better understanding of the factors that influence CNN system performance in healthcare applications. They offer practical insights for researchers and practitioners, emphasizing the importance of carefully choosing dataset sizes and employing data-balancing techniques to optimize model accuracy and reliability. It is recommended that practitioners focus on acquiring balanced datasets whenever possible for healthcare CNN tasks, even if this involves smaller dataset sizes. Moreover, it is advisable to consider the saturation point for dataset size and carefully assess the trade-off between performance gains and computational resources.

While this study provides valuable insights, it is essential to acknowledge its limitations. The study focused on specific healthcare applications and datasets; further investigations on different tasks and datasets may yield additional insights. Additionally, the study primarily examined the impact of dataset size and balance ratio, leaving room for exploring the interplay with other factors, such as feature engineering and model architecture.

In conclusion, this study advances CNN applications in healthcare by offering evidence-based guidelines for dataset selection and management. The unique examination of three-class classification and the insights gained can aid researchers and practitioners in developing more effective and robust CNN systems for healthcare-related tasks, ultimately enhancing the quality and efficiency of healthcare services.

## Figures and Tables

**Table 1:** Detailed architecture of the Convolutional Neural Network (CNN).

Layer (Type)	Output Shape	Param #
Conv2D	222, 222, 32	320
MaxPooling2D	111, 111, 32	0
Conv2D	109, 109, 64	18,496
MaxPooling2D	54, 54, 64	0
Conv2D	52, 52, 64	36,928
MaxPooling2D	26, 26, 64	0
Conv2D	24, 24, 128	73,856
MaxPooling2D	12, 12, 128	0
Conv2D	10, 10, 128	147,584
Batch_Normalization	10, 10, 128	512
Flatten	12,800	0
Dense	128	1,638,528
Batch_Normalization	128	512
Dropout	128	0
Dense	2	258
Total params: 1,916,994 Trainable params: 1,916,482 Non-trainable params: 512		

**Table 2:** The percentage ratios tested.

# of Classes	Percentage Division between Classes							
Two classes	50:50	60:40	70:30	80:20	90:10	95:5	99:1	
Three classes	49:49:2	45:45:10	40:40:20	33:33:33	60:20:20	80:10:10	90:5:5	98:1:1

**Table 3:** The tests conducted for binary classification.

Task	Binary Classification											
Split Method	Test 500 Images per Class						20% of the Data for Test					
	Fix			Change			Fix			Change		
Dataset Size	100 *	200 *	500 *	1000	5000	10,000	100 *	200 *	500 *	1000	5000	10,000
IR (%)												
50:50	V	V	V	V	V	∅	V	V	V	V	V	∅
60:40	V	V	V	V	V	∅	V	V	V	V	V	∅
70:30	V	V	V	V	V	V	V	V	V	V	V	V
80:20	V	V	V	V	V	V	V	V	V	V	V	V
90:10	V	V	V	V	V	V	V	V	V	V	V	V
95:5	V	V	V	V	V	V	V	V	V	V	V	V
98:2	V	V	∅	V	V	V	V	V	∅	V	V	V
99:1	V	∅	∅	V	V	V	V	∅	∅	V	V	V

V—Experiments performed. ∅—Experiments not performed due to required dataset size. * Images for COVID class.

**Table 4:** The tests conducted for classification of three classes.

Task	Three-Class Classification					
Split Method	Test 500 Images per Class			20% of the Data for Test		
	Change Size			Change Size		
Dataset Size	1000	5000	10,000	1000	5000	10,000
IR tested (%)	(1.0, 49.5, 49.5)	(5.0, 47.5, 47.5)	(20.0, 40.0, 40.0)		(80.0, 10.0, 10.0)	(95.0, 2.5, 2.5)
	(2.0, 49.0, 49.0)	(10.0, 45.0, 45.0)	(33.3, 33.3, 33.3)		(90.0, 5.0, 5.0)	(98.0, 1.0, 1.0)

## Data Availability

The original contributions presented in the study are included in the article/Supplementary Material. Further inquiries can be directed to the corresponding author.

## Supplementary Materials

The following supporting information can be downloaded at: https://www.mdpi.com/article/10.3390/diagnostics14161727/s1, Explanation of the indices that were used to evaluate the CNN model. Table S1. Test 20%, Fix, COVID size = 100; Table S2. Test 20%, Fix, COVID size = 200; Table S3. Test 20%, Fix, COVID size = 500; Table S4. Test 20%, Change, Total 1000; Table S5. Test 20%, Change, Total 5000; Table S6. Test 20%, Change, Total 10,000; Table S7. 500 Test, Fix, COVID size = 100; Table S8. 500 Test, Fix, COVID size = 200; Table S9. 500 Test, Fix, COVID size = 500; Table S10. 500 Test, Change, Total 1000; Table S11. 500 Test, Change, Total 5000; Table S12. 500 Test, Change, Total 10,000; Table S13. Test 20%, Change, Total 1000; Table S14. Test 20%, Change, Total 5000; Table S15. Test 20%, Change, Total 10,000; Table S16. 500 test, Change, Total 1000; Table S17. 500 test, Change, Total 5000; Table S18. 500 test, Change, Total 10,000; Figure S1. Confusion matrix. Test 20%, Change, Total 1000; Figure S2. Confusion matrix. Test 20%, Change, Total 5000; Figure S3. Confusion matrix. Test 20%, Change, Total 10,000; Figure S4. Confusion matrix. 500 test, Change, Total 1000; Figure S5. Confusion matrix. 500 test, Change, Total 5000; Figure S6. Confusion matrix. 500 test, Change, Total 10,000.

## References

[1] Rout N., Mishra D., Mallick M.K. (2018). Handling imbalanced data: A survey. International Proceedings on Advances in Soft Computing, Intelligent Systems and Applications.

[2] López V., Fernández A., García S., Palade V., Herrera F. (2013). An insight into classification with imbalanced data: Empirical results and current trends on using data intrinsic characteristics. Inf. Sci..

[3] Li Q., Mao Y. (2014). A review of boosting methods for imbalanced data classification. Pattern Anal. Appl..

[4] Han C., Wang P., Huang R., Cui L. (2022). HCTNet: An experience-guided deep learning network for inter-patient arrhythmia classification on imbalanced dataset. Biomed. Signal Process. Control.

[5] Li D.-C., Hu S.C., Lin L.-S., Yeh C.-W. (2017). Detecting representative data and generating synthetic samples to improve learning accuracy with imbalanced data sets. PLoS ONE.

[6] Lee Z.-J., Yang M.-R., Hwang B.-J. (2024). A Sustainable Approach to Asthma Diagnosis: Classification with Data Augmentation, Feature Selection, and Boosting Algorithm. Diagnostics.

[7] Alsalatie M., Alquran H., Mustafa W.A., Zyout A., Alqudah A.M., Kaifi R., Qudsieh S. (2023). A New Weighted Deep Learning Feature Using Particle Swarm and Ant Lion Optimization for Cervical Cancer Diagnosis on Pap Smear Images. Diagnostics.

[8] Li D., Zheng C., Zhao J., Liu Y. (2023). Diagnosis of heart failure from imbalance datasets using multi-level classification. Biomed. Signal Process. Control.

[9] Lu W., Hou H., Chu J. (2018). Feature fusion for imbalanced ECG data analysis. Biomed. Signal Process. Control.

[10] Vijayvargiya A., Prakash C., Kumar R., Bansal S., Tavares J.M.R. (2021). Human knee abnormality detection from imbalanced sEMG data. Biomed. Signal Process. Control.

[11] Rath A., Mishra D., Panda G., Satapathy S.C. (2021). Heart disease detection using deep learning methods from imbalanced. Biomed. Signal Process. Control.

[12] Hancer E., Samet M.T.R., Yıldırım Z., Nemati N. (2023). An imbalance-aware nuclei segmentation methodology for H&E stained. Biomed. Signal Process. Control.

[13] Arshad S., Amjad T., Hussain A., Qureshi I., Abbas Q. (2023). Dermo-Seg: ResNet-UNet Architecture and Hybrid Loss Function for Detection of Differential Patterns to Diagnose Pigmented Skin Lesions. Diagnostics.

[14] Wei Q., Dunbrack R.L. (2013). The role of balanced training and testing data sets for binary classifiers in bioinformatics. PLoS ONE.

[15] Kaur H., Pannu H.S., Malhi A.K. (2019). A systematic review on imbalanced data challenges in machine learning: Applications and solutions. ACM Comput. Surv. (CSUR).

[16] Mazurowskia M.A., Habasa P.A., Zuradaa J.M., Lob J.Y., Bakerb J.A., Tourassib G.D. (2008). Training neural network classifiers for medical decision making: The effects of imbalanced datasets on classification performance. Neural Netw..

[17] Bartosz K. (2016). Learning from imbalanced data: Open challenges and future directions. Prog. Artif. Intell..

[18] Sun Y., Kamel M.S., Wang Y. Boosting for learning multiple classes with imbalanced class distribution. Proceedings of the Sixth International Conference on Data Mining (ICDM’06).

[19] Pei X., Zhao Y.H., Chen L., Guo Q.D.Z., Pan Y., Hou H. (2023). Robustness of machine learning to color, size change, normalization, and image enhancement on micrograph datasets with large sample differences. Mater. Des..

[20] Bailly A., Blanc C., Francis É., Guillotin T., Jamal F., Wakim B., Roy P. (2022). Effects of dataset size and interactions on the prediction performance of logistic regression and deep learning models. Comput. Methods Programs Biomed..

[21] Choi W., Lee S. (2023). Performance evaluation of deep learning architectures for load and temperature forecasting under dataset size constraints and seasonality. Energy Build..

[22] He K., Zhang X., Ren S., Sun J. (2016). Identity Mappings in Deep Residual Networks. Computer Vision—ECCV 2016, Proceedings of the 14th European Conference, Amsterdam, The Netherlands, 11–14 October 2016.

[23] Huang G., Liu Z., Maaten L.V.D., Weinberger K.Q. Densely Connected Convolutional Networks. Proceedings of the IEEE Conference on Computer Vision and Pattern Recognition (CVPR).

[24] Hasib K.M., Azam S., Karim A., Marouf A.A., Shamrat F.M.J.M., Montaha S., Yeo K.C., Jonkman M., Alhajj R., Rokne J.G. (2023). CNN-LSTM: Combining CNN and LSTM to Classify Multi-Class Text in Imbalanced News Data. IEEE Access.

[25] Londhe M. (2021). Classification of Eye Diseases Using Hybrid CNN-RNN Models. Ph.D. These.

[26] Kotsiantis S.B., Pintelas P.E. (2007). Combining bagging and boosting. Int. J. Math. Comput. Sci..

[27] Kalirane M. (2024). Ensemble Learning in Machine Learning: Stacking, Bagging and Boosting. Analytics Vidhya. https://www.analyticsvidhya.com/blog/2023/01/ensemble-learning-methods-bagging-boosting-and-stacking/.

[28] Hasib K., Iqbal S., Shah F.M., Mahmud J.A., Popel M.H., Showrov I.H., Ahmed S., Rahman O. (2020). A Survey of Methods for Managing the Classification and Solution of Data Imbalance Problem. J. Comput. Sci..

[29] Li Z., Chen Q., Feng L., Rodewald L., Xia Y., Yu H., Zhang R., An Z., Yin W., Chen W. (2020). Active case finding with case management: The key to tackling the COVID-19 pandemic. The Lancet.

[30] Gandhi R.T., Lynch J.B., Del Rio C. (2020). Mild or moderate COVID-19. N. Engl. J. Med..

[31] Berlin D.A., Gulick R.M., Martinez F.J. (2020). Severe COVID-19. N. Engl. J. Med..

[32] Peeling R.W., Heymann D.L., Teo Y.Y., Garcia P.J. (2022). Diagnostics for COVID-19: Moving from pandemic response to control. The Lancet.

[33] Alsharif W., Qurashi A. (2021). Effectiveness of COVID-19 diagnosis and management tools: A review. Radiography.

[34] Ebrahimzadeh S., Islam N., Dawit H., Salameh J., Kazi S., Fabiano N., Treanor L., Absi M., Ahmad F., Rooprai P. (2022). Thoracic imaging tests for the diagnosis of COVID-19. Cochrane Database Syst. Rev..

[35] Jacobi A., Chung M., Bernheim A., Eber C. (2020). Portable chest X-ray in coronavirus disease-19 (COVID-19): A pictorial review. Clin. Imaging.

[36] Saygılı A. (2021). A new approach for computer-aided detection of coronavirus (COVID-19) from CT and X-ray images using machine learning methods. Appl. Soft Comput..

[37] Nur A.-A., Ahsan M., Based M.A., Haider J., Kowalski M. (2021). COVID-19 Detection from Chest X-ray Images Using Feature Fusion and Deep Learning. Sensors.

[38] Rahman T., Chowdhury M., Khandakar A. (2021). COVID-19 Radiography Database. https://www.kaggle.com/datasets/tawsifurrahman/covid19-radiography-database.

