# Supplementary material for: Exploring the Interplay of Dataset Size and Imbalance on CNN Performance in Healthcare: Using X-rays to Identify COVID-19 Patients
Source: Diagnostics (Basel). 2024 Aug 8;14(16):1727. doi: 10.3390/diagnostics14161727 (PMC11353409; doi:10.3390/diagnostics14161727)
Supplement: Supplementary file 1 [file diagnostics-14-01727-s001.zip › Supplementary Material File S1.pdf]

Supplementary Material File S1

Explanation of the indices that were used to evaluate the CNN model:

- **Sensitivity (Recall)**: This measure refers to the probability of a positive test, conditioned on truly being positive. It is sometimes also called as True Positive Rate (TPR). In our study, sensitivity was defined as the proportion of people classified as positive for COVID and actually had COVID among all those who actually had COVID.
- **Specificity:** This measure refers to the probability of a negative test, conditioned on truly being negative. It is sometimes also called as True Negative Rate (TNR). In our study, specificity was defined as the proportion of people who test negative for COVID and actually healthy people among those who are really healthy.
- **Precision**: This measure is defined as the ratio of true positives (TP) to the total number of positive examples predicted.
- **F-measure/F1 score**: This measure is the harmonic mean of precision and sensitivity. The highest possible value of an F1 score is 1.0, indicating perfect precision and sensitivity, and the lowest possible value is 0, if either the precision or the sensitivity is zero. The F-score is also used for evaluating classification problems with more than two classes. In this study, we used macro-averaging, the arithmetic mean of classes' F1 scores.
- **G-measure**: This measure is the geometric mean that evaluated as the square root of multiplication of sensitivity and specificity. Used to measure the balance between classification performances on both the majority and minority classes. A low score indicates poor performance in classifying the positive cases even if the negative cases are successfully classified [22].

- **AUC-ROC**: The Receiver Operating Curve (ROC) visually depicts the difference between accuracy for positive examples and error for negative examples, where AUC is the abbreviation for Area under Curve. This curve evaluates trade-offs between true positives and false positives in respect to the limit of threshold of a predictive model. The AUC value varies between 0 and 1, with the worst performance marked as 0 and the best performance as 1. The model is classified as best if it obtains True Positive Rate and False Positive Rate as 1 and 0, respectively.

In our study we used ‘Sklearn’, useful library for machine learning in Python, to evaluate the classification measure.
