# Supplementary material for: Exploring the Interplay of Dataset Size and Imbalance on CNN Performance in Healthcare: Using X-rays to Identify COVID-19 Patients
Source: Diagnostics (Basel). 2024 Aug 8;14(16):1727. doi: 10.3390/diagnostics14161727 (PMC11353409; doi:10.3390/diagnostics14161727)
Supplement: Supplementary file 1 [file diagnostics-14-01727-s001.zip › Supplementary Material File S2.pdf]

Supplementary Material File S2

Tables S1–S3: The Results for the Split-Data Method in Which the Amount of Data of the COVID Class Is Constant (Fix) 100, 200, and 500 Images.

Table S1. Test 20%, Fix, COVID size = 100

Ratio	Ratio %	Accuracy	Sensitivity	Specificity	Precision	F1 score	G measure	AUC	TN	FP	FN	TP
**(100, 100)**	(50, 50)	0.675	0.6	0.75	0.705	0.648	0.670	0.675	15	5	8	12
**(150, 100)**	(60, 40)	0.56	0.75	0.433	0.468	0.576	0.570	0.591	13	17	5	15
**(233, 100)**	(70, 30)	0.701	0.05	0.978	0.5	0.09	0.221	0.514	46	1	19	1
**(400, 100)**	(80, 20)	0.78	0.05	0.962	0.25	0.083	0.219	0.506	77	3	19	1
**(900, 100)**	(90, 10)	0.885	0.05	0.977	0.2	0.08	0.221	0.513	176	4	19	1
**(1900, 100)**	(95, 5)	0.952	0.05	1	1	0.095	0.223	0.525	380	0	19	1
**(4900, 100)**	(98, 2)	0.98	0	1			0	0.5	980	0	20	0
**(9900, 100)**	(99, 1)	0.99	0	1			0	0.5	1980	0	20	0

Table S2. Test 20%, Fix, COVID size = 200

Ratio	Ratio %	Accuracy	Sensitivity	Specificity	Precision	F1 score	G measure	AUC	TN	FP	FN	TP
**(200, 200)**	(50, 50)	0.7125	0.975	0.45	0.639	0.772	0.662	0.7125	18	22	1	39
**(300, 200)**	(60, 40)	0.76	0.675	0.816	0.71	0.692	0.742	0.745	49	11	13	27
**(467, 200)**	(70, 30)	0.761	0.5	0.872	0.625	0.555	0.660	0.686	82	12	20	20
**(800, 200)**	(80, 20)	0.795	0.525	0.862	0.488	0.506	0.672	0.693	138	22	19	21
**(1800, 200)**	(90, 10)	0.905	0.1	0.994	0.666	0.173	0.315	0.547	358	2	36	4
**(3800, 200)**	(95, 5)	0.95	0	1			0	0.5	760	0	40	0
**(9800, 200)**	(98, 2)	0.98	0	1			0	0.5	1960	0	40	0

Table S3. Test 20%, Fix, COVID size = 500

Ratio	Ratio %	Accuracy	Sensitivity	Specificity	Precision	F1 score	G measure	AUC	TN	FP	FN	TP
**(500, 500)**	(50, 50)	0.77	0.84	0.7	0.737	0.785	0.767	0.77	70	30	16	84
**(750, 500)**	(60, 40)	0.76	0.82	0.72	0.661	0.732	0.768	0.77	108	42	18	82
**(1165, 500)**	(70, 30)	0.874	0.74	0.931	0.822	0.779	0.83	0.836	217	16	26	74
**(2000, 500)**	(80, 20)	0.894	0.81	0.915	0.704	0.753	0.861	0.862	366	34	19	81
**(4500, 500)**	(90, 10)	0.906	0.1	0.996	0.714	0.175	0.315	0.548	896	4	90	10
**(9500, 500)**	(95, 5)	0.9615	0.3	0.996	0.811	0.438	0.547	0.648	1893	7	70	30

Tables S4–S6: The Results for the Split-Data Method Where the Amount of Training Data for the Two Classes Changes (Change) but the Sum of the Data of the two Classes Is Constant for total data are 1000, 5000, and 10000 images

Table S4. Test 20%, Change, Total 1000

Ratio	Ratio %	Accuracy	Sensitivity	Specificity	Precision	F1 score	G measure	AUC	TN	FP	FN	TP
**(500, 500)**	(50, 50)	0.8	0.8	0.8	0.8	0.8	0.8	0.8	80	20	20	80
**(600, 400)**	(60, 40)	0.745	0.512	0.9	0.773	0.616	0.679	0.706	108	12	39	41
**(700, 300)**	(70, 30)	0.79	0.366	0.971	0.846	0.511	0.596	0.669	136	4	38	22
**(800, 200)**	(80, 20)	0.735	0.175	0.875	0.259	0.208	0.391	0.525	140	20	33	7
**(900, 100)**	(90, 10)	0.895	0	0.994	0		0	0.497	179	1	20	0
**(950, 50)**	(95, 5)	0.925	0.4	0.952	0.307	0.347	0.617	0.676	181	9	6	4
**(980, 20)**	(98, 2)	0.975	0	0.994	0		0	0.497	195	1	4	0
**(990, 10)**	(99, 1)	0.99	0	1			0	0.5	198	0	2	0

Table S5. Test 20%, Change, Total 5000

Ratio	Ratio %	Accuracy	Sensitivity	Specificity	Precision	F1 score	G measure	AUC	TN	FP	FN	TP
**(2500, 2500)**	(50, 50)	0.94	0.954	0.926	0.928	0.94	0.939	0.94	463	37	23	477
**(3000, 2000)**	(60, 40)	0.953	0.96	0.948	0.925	0.942	0.954	0.954	569	31	16	384
**(3500, 1500)**	(70, 30)	0.932	0.84	0.971	0.926	0.881	0.903	0.905	680	20	48	252
**(4000, 1000)**	(80, 20)	0.926	0.75	0.97	0.862	0.802	0.852	0.86	776	24	50	150
**(4500, 500)**	(90, 10)	0.912	0.22	0.988	0.687	0.333	0.466	0.604	890	10	78	22
**(4750, 250)**	(95, 5)	0.953	0.08	0.998	0.8	0.145	0.282	0.539	949	1	46	4
**(4900, 100)**	(98, 2)	0.98	0	1			0	0.5	980	0	20	0
**(4950, 50)**	(99, 1)	0.989	0	0.998	0		0	0.499	989	1	10	0

Table S6. Test 20%, Change, Total 10000

Ratio	Ratio %	Accuracy	Sensitivity	Specificity	Precision	F1 score	G measure	AUC	TN	FP	FN	TP
**(7000, 3000)**	(70, 30)	0.955	0.94	0.961	0.912	0.926	0.950	0.95	1346	54	36	564
**(8000, 2000)**	(80, 20)	0.967	0.925	0.978	0.913	0.919	0.951	0.951	1565	35	30	370
**(9000, 1000)**	(90, 10)	0.954	0.61	0.992	0.897	0.726	0.777	0.801	1786	14	78	122
**(9500, 500)**	(95, 5)	0.969	0.49	0.994	0.816	0.612	0.697	0.742	1889	11	51	49
**(9800, 200)**	(98, 2)	0.979	0.05	0.998	0.4	0.088	0.223	0.524	1957	3	38	2
**(9900, 100)**	(99, 1)	0.989	0	0.999	0		0	0.499	1979	1	20	0

Tables S7–S9: The Results for the Split-Data Method in Which the Amount of Data for the COVID Class Is Constant (Fix) for 100, 200, And 500 Images

Table S7. 500 Test, Fix, COVID size = 100

Ratio	Ratio %	Accuracy	Sensitivity	Specificity	Precision	F1 score	G measure	AUC	TN	FP	FN	TP
**(100, 100)**	(50, 50)	0.659	0.62	0.698	0.672	0.645	0.657	0.659	349	151	190	310
**(150, 100)**	(60, 40)	0.648	0.522	0.774	0.697	0.597	0.635	0.648	387	113	239	261
**(233, 100)**	(70, 30)	0.631	0.406	0.856	0.738	0.523	0.589	0.631	428	72	297	203
**(400, 100)**	(80, 20)	0.611	0.286	0.936	0.817	0.423	0.517	0.611	468	32	357	143
**(900, 100)**	(90, 10)	0.59	0.208	0.972	0.881	0.336	0.449	0.59	486	14	396	104
**(1900, 100)**	(95, 5)	0.544	0.088	1	1	0.161	0.296	0.544	500	0	456	44
**(4900, 100)**	(98, 2)	0.54	0.08	1	1	0.148	0.282	0.54	500	0	460	40
**(9900, 100)**	(99, 1)	0.542	0.088	0.996	0.956	0.161	0.296	0.542	498	2	456	44

Table S8. 500 Test, Fix, COVID size = 200

Ratio	Ratio %	Accuracy	Sensitivity	Specificity	Precision	F1 score	G measure	AUC	TN	FP	FN	TP
**(200, 200)**	(50, 50)	0.67	0.634	0.706	0.683	0.657	0.669	0.67	353	147	183	317
**(300, 200)**	(60, 40)	0.678	0.506	0.85	0.771	0.611	0.655	0.678	425	75	247	253
**(467, 200)**	(70, 30)	0.619	0.252	0.986	0.947	0.398	0.498	0.619	493	7	374	126
**(800, 200)**	(80, 20)	0.636	0.306	0.966	0.9	0.456	0.543	0.636	483	17	347	153
**(1800, 200)**	(90, 10)	0.626	0.26	0.992	0.97	0.41	0.507	0.626	496	4	370	130
**(3800, 200)**	(95, 5)	0.562	0.124	1	1	0.22	0.352	0.562	500	0	438	62
**(9800, 200)**	(98, 2)	0.591	0.184	0.998	0.989	0.31	0.428	0.591	499	1	408	92

Table S9. 500 Test, Fix, COVID size = 500

Ratio	Ratio %	Accuracy	Sensitivity	Specificity	Precision	F1 score	G measure	AUC	TN	FP	FN	TP
**(500, 500)**	(50, 50)	0.787	0.744	0.83	0.814	0.777	0.785	0.787	415	85	128	372
**(750, 500)**	(60, 40)	0.781	0.626	0.936	0.907	0.74	0.765	0.781	468	32	187	313
**(1165, 500)**	(70, 30)	0.822	0.708	0.936	0.917	0.799	0.814	0.822	468	32	146	354
**(2000, 500)**	(80, 20)	0.729	0.476	0.982	0.963	0.637	0.683	0.729	491	9	262	238
**(4500, 500)**	(90, 10)	0.781	0.6	0.962	0.94	0.732	0.759	0.781	481	19	200	300
**(9500, 500)**	(95, 5)	0.697	0.4	0.994	0.985	0.568	0.630	0.697	497	3	300	200

Tables S10–S12: The Results for the Split-Data Method Where the Amount of Data for the Two Classes Changes (Change) but the Sum of the Data for the Two Classes Is Constant for Cases Where the Total Data Are 1000, 5000, And 10,000 Images

Table S10. 500 Test, Change, Total 1000

Ratio	Ratio %	Accuracy	Sensitivity	Specificity	Precision	F1 score	G measure	AUC	TN	FP	FN	TP
**(500, 500)**	(50, 50)	0.751	0.592	0.91	0.868	0.703	0.733	0.751	455	45	204	296
**(600, 400)**	(60, 40)	0.827	0.746	0.908	0.89	0.811	0.823	0.827	454	46	127	373
**(700, 300)**	(70, 30)	0.754	0.592	0.916	0.875	0.706	0.736	0.754	458	42	204	296
**(800, 200)**	(80, 20)	0.702	0.492	0.912	0.848	0.622	0.669	0.702	456	44	254	246
**(900, 100)**	(90, 10)	0.553	0.118	0.988	0.907	0.208	0.341	0.553	494	6	441	59
**(950, 50)**	(95, 5)	0.555	0.126	0.984	0.887	0.22	0.352	0.555	492	8	437	63
**(980, 20)**	(98, 2)	0.515	0.034	0.996	0.894	0.065	0.184	0.515	498	2	483	17
**(990, 10)**	(99, 1)	0.5	0	1			0	0.5	500	0	500	0

Table S11. 500 Test, Change, Total 5000

Ratio	Ratio %	Accuracy	Sensitivity	Specificity	Precision	F1 score	G measure	AUC	TN	FP	FN	TP
**(2500, 2500)**	(50, 50)	0.925	0.894	0.956	0.953	0.922	0.924	0.925	478	22	53	447
**(3000, 2000)**	(60, 40)	0.92	0.882	0.958	0.954	0.916	0.919	0.92	479	21	59	441
**(3500, 1500)**	(70, 30)	0.934	0.922	0.946	0.944	0.933	0.933	0.934	473	27	39	461
**(4000, 1000)**	(80, 20)	0.897	0.824	0.97	0.964	0.888	0.894	0.897	485	15	88	412
**(4500, 500)**	(90, 10)	0.648	0.338	0.958	0.889	0.489	0.569	0.648	479	21	331	169
**(4750, 250)**	(95, 5)	0.666	0.344	0.988	0.966	0.507	0.582	0.666	494	6	328	172
**(4900, 100)**	(98, 2)	0.555	0.112	0.998	0.982	0.201	0.334	0.555	499	1	444	56
**(4950, 50)**	(99, 1)	0.501	0.002	1	1	0.003	0.044	0.501	500	0	499	1

Table S12. 500 Test, Change, Total 10000

Ratio	Ratio %	Accuracy	Sensitivity	Specificity	Precision	F1 score	G measure	AUC	TN	FP	FN	TP
**(7000, 3000)**	(70, 30)	0.944	0.906	0.982	0.98	0.941	0.943	0.944	491	9	47	453
**(8000, 2000)**	(80, 20)	0.95	0.908	0.992	0.991	0.947	0.949	0.95	496	4	46	454
**(9000, 1000)**	(90, 10)	0.866	0.766	0.966	0.957	0.851	0.860	0.866	483	17	117	383
**(9500, 500)**	(95, 5)	0.705	0.422	0.988	0.972	0.588	0.645	0.705	494	6	289	211
**(9800, 200)**	(98, 2)	0.543	0.09	0.996	0.957	0.164	0.299	0.543	498	2	455	45
**(9900, 100)**	(99, 1)	0.526	0.052	1	1	0.0988	0.228	0.526	500	0	474	26

Tables S13–S15 and Figures 1-3: The Results for the Split-Data Method Where the Amount of Training Data for the Three Classes Changes (Change) but the Sum of the Data for All Classes Is Constant for Cases Where the Total Data Are 1000, 5000, and 10,000 Images

Table S13. Test 20%, Change, Total 1000

Ratio	Ratio %	Accuracy	Normal Sensitivity	COVID Sensitivity	Lung Opacity Sensitivity	Macro Avg Precision	Macro Avg F1-score
**(10, 495, 495)**	(1.0, 49.5, 49.5)	0.92	0.5	0.878	0.969	0.732	0.750
**(20, 490, 490)**	(2.0, 49.0, 49.0)	0.865	0.25	0.846	0.908	0.638	0.641
**(50, 475, 475)**	(5.0, 47.5, 47.5)	0.865	0.4	0.926	0.852	0.775	0.745
**(100, 450, 450)**	(10.0, 45.0, 45.0)	0.85	0.75	0.855	0.866	0.772	0.788
**(200, 400, 400)**	(20.0, 40.0, 40.0)	0.88	0.85	0.887	0.8875	0.858	0.863
**(333, 333, 333)**	(33.33, 33.33, 33.33)	0.855	0.865	0.895	0.805	0.857	0.855
**(600, 200, 200)**	(60.0, 20.0, 20.0)	0.795	0.775	0.95	0.7	0.750	0.771
**(800, 100, 100)**	(80.0, 10.0, 10.0)	0.86	0.987	0.15	0.55	0.749	0.616
**(900, 50, 50)**	(90.0, 5.0, 5.0)	0.86	0.894	0.2	0.9	0.750	0.564
**(950, 25, 25)**	(95.0, 2.5, 2.5)	0.95	1	0	0	0.316	0.324
**(980, 10, 10)**	(98.0, 1.0, 1.0)	0.975	0.994	0	0	0.326	0.329

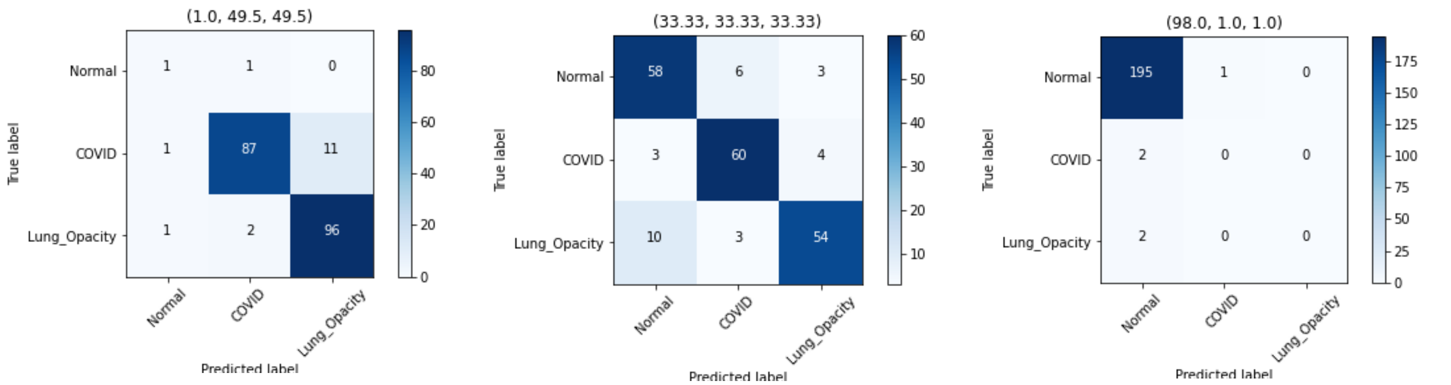

Figure 1. Confusion matrix. Test 20%, Change, Total 1000

Table S14. Test 20%, Change, Total 5000

Ratio	Ratio %	Accuracy	Normal Sensitivity	COVID Sensitivity	Lung Opacity Specificity	Macro Avg Precision	Macro Avg F1-score
**(50, 2475, 2475)**	(1.0, 49.5, 49.5)	0.906	0	0.903	0.893	0.605	0.607
**(100, 2450, 2450)**	(2.0, 49.0, 49.0)	0.898	0.2	0.867	0.852	0.717	0.689
**(250, 2375, 2375)**	(5.0, 47.5, 47.5)	0.873	0.44	0.901	0.876	0.746	0.745
**(500, 2250, 2250)**	(10.0, 45.0, 45.0)	0.836	0.58	0.828	0.82	0.777	0.771
**(1000, 2000, 2000)**	(20.0, 40.0, 40.0)	0.859	0.66	0.912	0.866	0.850	0.835
**(1666, 1666, 1666)**	(33.33, 33.33, 33.33)	0.869	0.856	0.955	0.955	0.870	0.868
**(3000, 1000, 1000)**	(60.0, 20.0, 20.0)	0.852	0.868	0.825	0.9075	0.821	0.829
**(4000, 500, 500)**	(80.0, 10.0, 10.0)	0.897	0.971	0.58	0.977	0.848	0.773
**(4500, 250, 250)**	(90.0, 5.0, 5.0)	0.91	0.971	0.64	0.993	0.638	
**(4750, 125, 125)**	(95.0, 2.5, 2.5)	0.95	0.995	0.16	0.997	0.540	
**(4900, 50, 50)**	(98.0, 1.0, 1.0)	0.98	1	0	1	0.326	

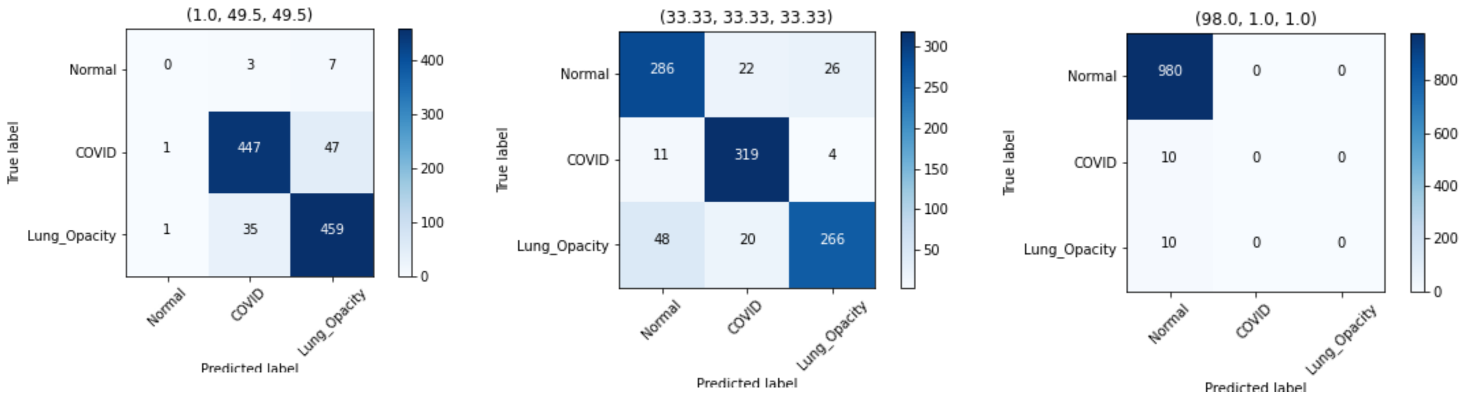

Figure 2. Confusion matrix. Test 20%, Change, Total 5000

Table S15. Test 20%, Change, Total 10000

Ratio	Ratio %	Accuracy	Normal Sensitivity	COVID Sensitivity	Lung Opacity Sensitivity	Macro Avg Precision	Macro Avg F1-score
**(100, 4950, 4950)**	(1.0, 49.5, 49.5)	0.860	0.1	0.809	0.927	0.674	0.626
**(200, 4900, 4900)**	(2.0, 49.0, 49.0)	0.858	0.2	0.783	0.959	0.732	0.669
**(500, 4750, 4750)**	(5.0, 47.5, 47.5)	0.87	0.37	0.867	0.925	0.751	0.732
**(1000, 4500, 4500)**	(10.0, 45.0, 45.0)	0.851	0.705	0.84	0.894	0.796	0.802
**(2000, 4000, 4000)**	(20.0, 40.0, 40.0)	0.858	0.745	0.886	0.887	0.849	0.844
**(3333, 3333, 3333)**	(33.33, 33.33, 33.33)	0.876	0.842	0.935	0.851	0.876	0.876
**(6000, 2000, 2000)**	(60.0, 20.0, 20.0)	0.875	0.888	0.917	0.792	0.847	0.856
**(8000, 1000, 1000)**	(80.0, 10.0, 10.0)	0.903	0.966	0.635	0.66	0.825	0.785
**(9000, 500, 500)**	(90.0, 5.0, 5.0)	0.938	0.983	0.52	0.54	0.809	0.733
**(9500, 250, 250)**	(95.0, 2.5, 2.5)	0.950	0.995	0.02	0.16	0.619	0.420
**(9800, 100, 100)**	(98.0, 1.0, 1.0)	0.98	1	0	0	0.326	0.329

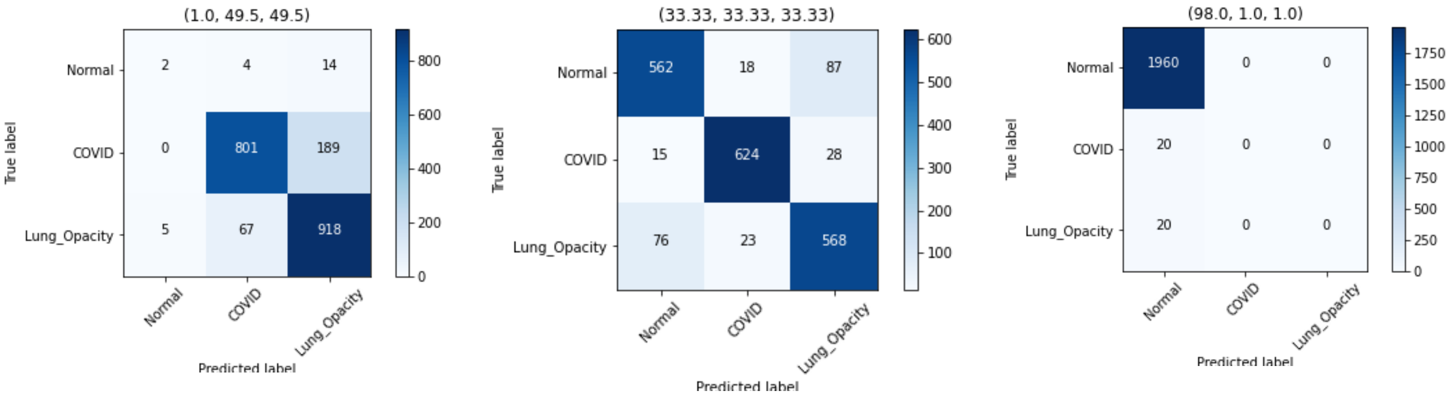

Figure 3. Confusion matrix. Test 20%, Change, Total 10000

Tables S16–S18 and Figures 4-6: The Split-Data Method Where the Amount of Data for the Three Classes Changes (Change) but the Sum of the Data for All Classes Is Constant for Cases Where the Total Data Are 1000, 5000, and 10,000 Images

Table S16. 500 test, Change, Total 1000

Ratio	Ratio %	Accuracy	Normal Sensitivity	COVID Sensitivity	Lung Opacity Sensitivity	Macro Avg Precision	Macro Avg F1-score
**(10, 495, 495)**	(1.0, 49.5, 49.5)	0.658	0.098	0.944	0.934	0.748	0.576
**(20, 490, 490)**	(2.0, 49.0, 49.0)	0.72	0.262	0.952	0.948	0.779	0.677
**(50, 475, 475)**	(5.0, 47.5, 47.5)	0.686	0.174	0.942	0.942	0.759	0.622
**(100, 450, 450)**	(10.0, 45.0, 45.0)	0.806	0.598	0.926	0.896	0.818	0.800
**(200, 400, 400)**	(20.0, 40.0, 40.0)	0.819	0.6	0.954	0.904	0.855	0.852
**(333, 333, 333)**	(33.33, 33.33, 33.33)	0.850	0.886	0.794	0.872	0.861	0.851
**(600, 200, 200)**	(60.0, 20.0, 20.0)	0.828	0.958	0.826	0.702	0.856	0.829
**(800, 100, 100)**	(80.0, 10.0, 10.0)	0.668	0.964	0.478	0.564	0.771	0.664
**(900, 50, 50)**	(90.0, 5.0, 5.0)	0.472	0.998	0.198	0.22	0.680	0.412
**(950, 25, 25)**	(95.0, 2.5, 2.5)	0.361	1	0.064	0.02	0.761	0.223
**(980, 10, 10)**	(98.0, 1.0, 1.0)	0.386	0.998	0.02	1	0.632	0.266

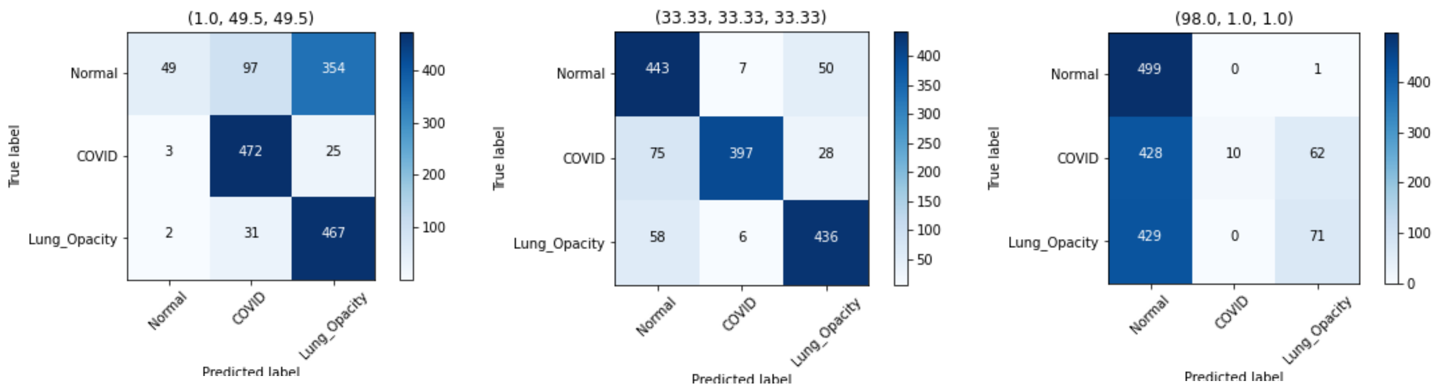

Figure 4. Confusion matrix. 500 test, Change, Total 1000

Table S17. 500 test, Change, Total 5000

Ratio	Ratio %	Accuracy	Normal Sensitivity	COVID Sensitivity	Lung Opacity Sensitivity	Macro Avg Precision	Macro Avg F1-score
**(50, 2475, 2475)**	(1.0, 49.5, 49.5)	0.657	0.066	0.932	0.974	0.767	0.564
**(100, 2450, 2450)**	(2.0, 49.0, 49.0)	0.646	0.034	0.968	0.936	0.753	0.538
**(250, 2375, 2375)**	(5.0, 47.5, 47.5)	0.72	0.282	0.95	0.93	0.788	0.681
**(500, 2250, 2250)**	(10.0, 45.0, 45.0)	0.852	0.688	0.97	0.9	0.859	0.848
**(1000, 2000, 2000)**	(20.0, 40.0, 40.0)	0.853	0.704	0.95	0.906	0.857	0.850
**(1666, 1666, 1666)**	(33.33, 33.33, 33.33)	0.876	0.838	0.906	0.886	0.877	0.876
**(3000, 1000, 1000)**	(60.0, 20.0, 20.0)	0.816	0.934	0.846	0.668	0.841	0.815
**(4000, 500, 500)**	(80.0, 10.0, 10.0)	0.658	0.974	0.452	0.548	0.780	0.654
**(4500, 250, 250)**	(90.0, 5.0, 5.0)	0.719	0.97	0.582	0.606	0.795	0.718
**(4750, 125, 125)**	(95.0, 2.5, 2.5)	0.391	0.998	0.14	0.036	0.692	0.278
**(4900, 50, 50)**	(98.0, 1.0, 1.0)	0.343	1	0.022	0.008	0.684	0.187

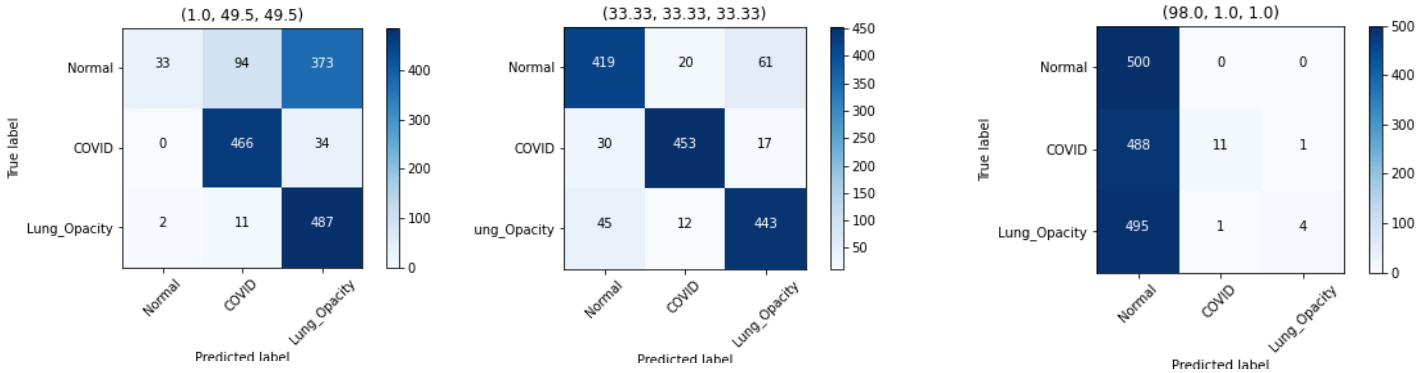

Figure 5. Confusion matrix. 500 test, Change, Total 5000

Table S18. 500 test, Change, Total 10000

Ratio	Ratio %	Accuracy	Normal Sensitivity	COVID Sensitivity	Lung Opacity Sensitivity	Macro Avg Precision	Macro Avg F1-score
**(100, 4950, 4950)**	(1.0, 49.5, 49.5)	0.664	0.11	0.894	0.988	0.741	0.594
**(200, 4900, 4900)**	(2.0, 49.0, 49.0)	0.688	0.14	0.96	0.966	0.790	0.618
**(500, 4750, 4750)**	(5.0, 47.5, 47.5)	0.724	0.246	0.97	0.956	0.797	0.674
**(1000, 4500, 4500)**	(10.0, 45.0, 45.0)	0.806	0.5	0.962	0.958	0.849	0.794
**(2000, 4000, 4000)**	(20.0, 40.0, 40.0)	0.857	0.676	0.966	0.93	0.869	0.853
**(3333, 3333, 3333)**	(33.33, 33.33, 33.33)	0.886	0.876	0.944	0.84	0.886	0.886
**(6000, 2000, 2000)**	(60.0, 20.0, 20.0)	0.838	0.932	0.88	0.704	0.856	0.838
**(8000, 1000, 1000)**	(80.0, 10.0, 10.0)	0.764	0.974	0.854	0.466	0.837	0.756
**(9000, 500, 500)**	(90.0, 5.0, 5.0)	0.617	0.97	0.286	0.596	0.749	0.595
**(9500, 250, 250)**	(95.0, 2.5, 2.5)	0.444	0.998	0.13	0.979	0.709	0.371
**(9800, 100, 100)**	(98.0, 1.0, 1.0)	0.349	1	0.048	0.998	0.420	0.199

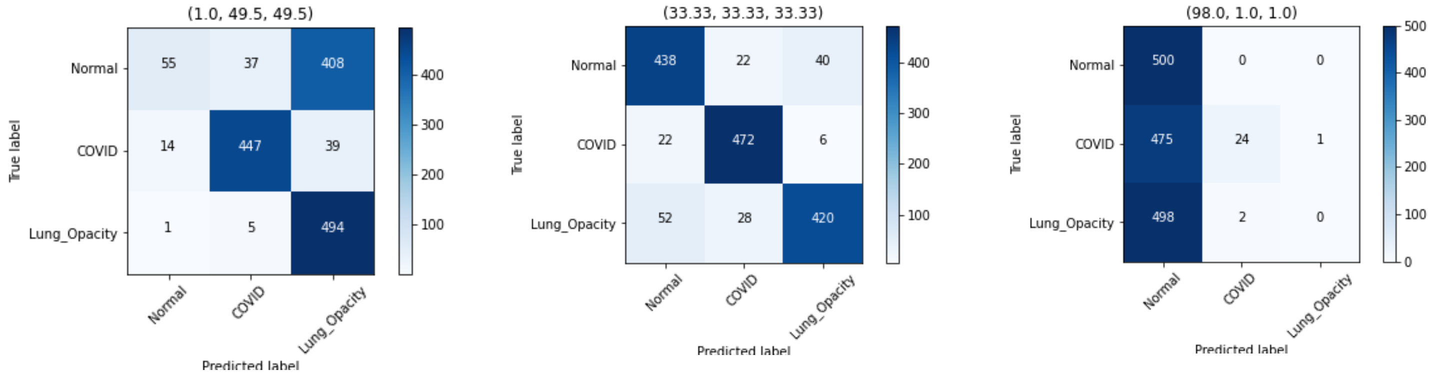

Figure 6. Confusion matrix. 500 test, Change, Total 10000
